# Case Report: The molecular fingerprint and the clinical implication of an exceptional response to neoadjuvant therapy in a metastatic cardia adenocarcinoma

**DOI:** 10.3389/fsurg.2024.1297083

**Published:** 2024-04-16

**Authors:** Laura Lorenzon, Andrea Campisi, Alessandra Di Paolo, Felice Giuliante, Fiamma Buttitta, Domenico D’Ugo

**Affiliations:** ^1^ Fondazione Policlinico Universitario Agostino Gemelli IRCCS, Catholic University of the Sacred Heart, Rome, Italy; ^2^ Department of Medical Oncology, Spirito Santo Hospital, Pescara, Italy; ^3^ Diagnostic Molecular Oncology Section, Center for Advanced Studies and Technology (CAST), University of Chieti-Pescara, Chieti-Pescara, Italy

**Keywords:** cardia adenocarcinoma, preoperative treatment, complete tumor regression, exceptional response, TP53

## Abstract

**Background:**

Globally, gastric cancer holds the fifth position in terms of prevalence among malignant tumors and is the fourth leading cause of cancer-related mortality. Particular attention should be paid to cardia adenocarcinoma (CA) due to its increasing incidence and poor prognosis. Diagnosis of CA frequently occurs in advanced stages because of its late symptoms. In such cases, neoadjuvant chemotherapy is the primary treatment option. The response to chemotherapy depends on multiple variables including the tumor's molecular profile, the patient's performance status, and the feasibility of using targeted therapy. Patients exhibiting an exceptional response, defined as a complete response to medical therapy lasting more than 1 year, or a partial response or stable disease lasting more than 2 years, are rarely described. This case report presents one of the longest-lasting exceptional responses to chemotherapy in metastatic cardia adenocarcinoma and discusses its clinical implications.

**Case presentation:**

A 49-year-old male patient presented with cardia adenocarcinoma (human epidermal growth factor receptor 2 negative, mismatch repair proficient) and liver metastases. Molecular profiling identified a pathogenic mutation in the TP53 gene (R123W; Arg123Trp) as the sole alteration found. Five months after initiating the neoadjuvant chemotherapy with fluorouracil–leucovorin–oxaliplatin–docetaxel, the patient achieved a complete clinical response. The molecular profile was compared with others previously documented in an international data portal, revealing a similar pattern. At 4 years and 3 months from diagnosis, the exceptional response was still confirmed. The patient underwent a cumulative number of 33 cycles of chemotherapy, leading to chemotherapy-induced liver damage.

**Conclusions:**

Exceptional responses to neoadjuvant chemotherapy in cardia adenocarcinomas are rarely reported. The documentation of exceptional responses to cancer therapies should be included in large data repositories to explore the molecular fingerprint of these tumors. In such cases, the clinical implications of long-term chemotherapy should always be taken into account.

## Introduction

Gastric cancer (GC) is globally ranked as the fifth most common malignant tumor and the fourth leading cause of cancer mortality (1).

Nowadays, particular attention should be paid to cardia adenocarcinoma (CA) due to its increasing incidence reported worldwide over the last five decades (2).

The five-year survival rate for CA currently stands at 10%–20%, significantly reduced for patients who do not undergo surgical resection, plummeting to 3%–5% (3). Indeed, the majority of CA cases present as advanced diseases, making neoadjuvant chemo/chemoradiotherapy the primary treatment option (4).

In case of locally advanced or metastatic disease, the recommended regimens for first-line systemic therapy include a fluoropyrimidine (fluorouracil or capecitabine) combined with either oxaliplatin or cisplatin (category 2B) (5).

More precisely, according to the latest European Society for Medical Oncology (ESMO) guidelines, fluorouracil–leucovorin–oxaliplatin–docetaxel (FLOT) is the regimen of protocol that leads to a higher rate of regression in locally advanced CA and it represents the preferred scheme of chemotherapy in patients capable of tolerating it (6).

Subsequently, GC may exhibit varying degrees of response, often documented as regression grades in the surgical specimen (7). In this context, a better clinical profile, human epidermal growth factor receptor 2 (HER2) expression, and intestinal histotype are characteristics often correlated with tumor regression (8). However, beyond pathologic regression, the definition of response can encompass a clinical outcome over time. Based on criterion, an exceptional response (ER) is defined as a complete response to medical therapy lasting more than 1 year, or a partial response or stable disease lasting more than 2 years (9).

Here we present one of the longest-lasting exceptional responses to chemotherapy in a cardia adenocarcinoma with liver metastases, along with its molecular fingerprint and clinical outcome. The molecular findings were also compared with those of other exceptional responders documented in the literature.

## Case presentation

The patient is a 49-year-old Caucasian male with CA and synchronous multiple liver metastasis. His oncological history began in June 2019, when he experienced asthenia, dyspepsia, and dysphagia with solids, along with a reported weight loss of 5 kg over the preceding three months. An esophagogastroduodenoscopy (EGDS) revealed an ulcerated neoplasm in the lower third of the esophagus, with circumferential thickening extending distally to the gastric lesser curve. A biopsy was performed and the histological examination was consistent with a poorly differentiated adenocarcinoma, HER2 negative, and presence of MLH1, MSH2, MSH6, PMS2 protein expression (microsatellite stable) on the immunohistochemistry (IHC) analyses.

Subsequent contrast-enhanced total body computed tomography (CT) and positron emission tomography (PET) scans documented a cT3 tumor with locoregional lymphadenopathy cN3, and multiple hepatic lesions (cM1).

The case was discussed during the Institutional multidisciplinary meeting, and it was decided to proceed with neoadjuvant therapy, which consisted of nine cycles of FLOT followed by three cycles of 5-florouracil and folinic acid (de Gramont scheme).

Remarkably, just 2 months after starting chemotherapy, a CT scan indicated an optimal response, as the thickening of the cardia was no longer evident, lymphadenopathy and liver metastases had significantly reduced in size, and a new EGDS showed a substantial reduction in the size of the tumor, now measuring 2 cm.

**Figure 1:**
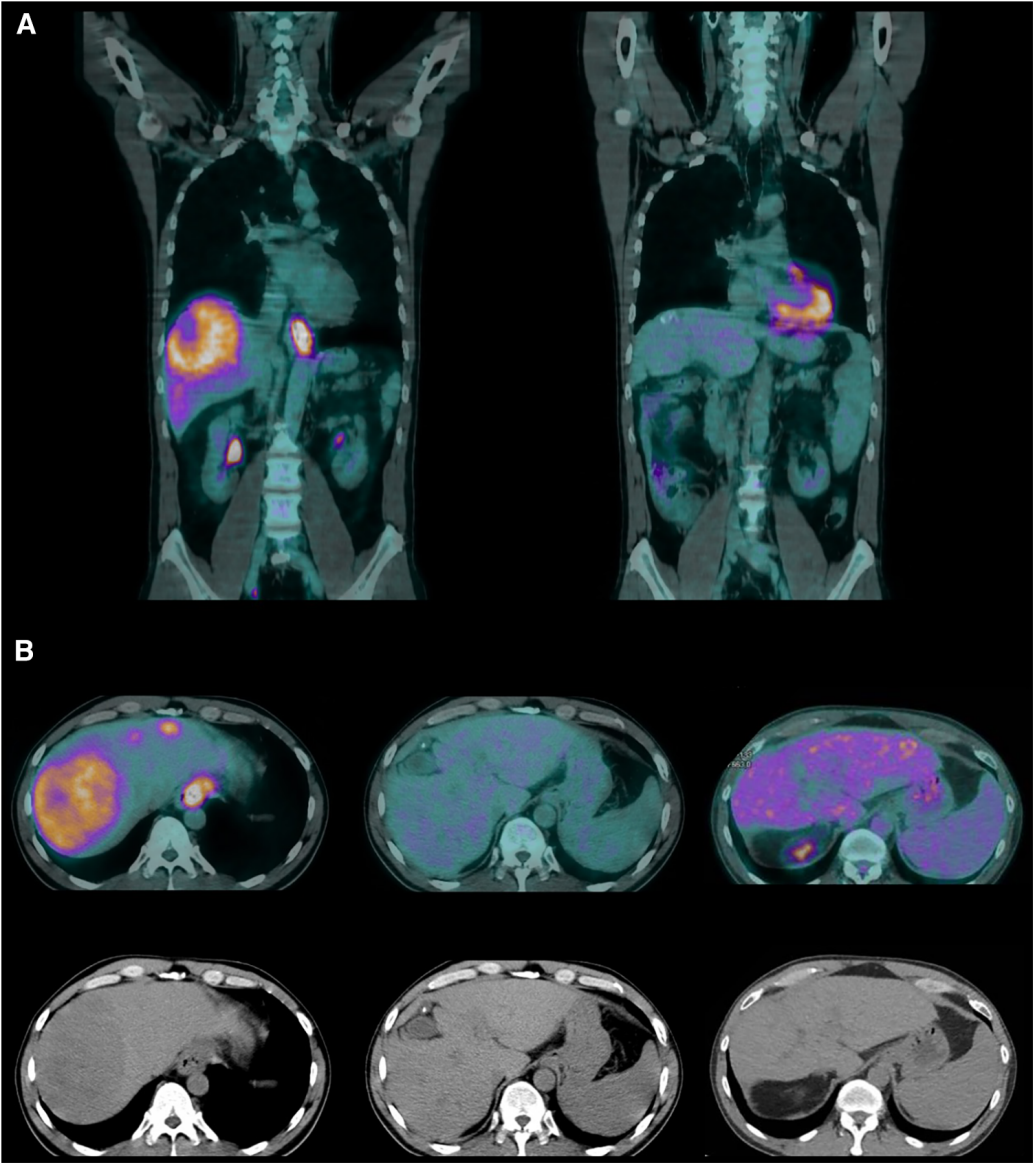
(**A**) Coronal PET scans (June 2019 and March 2021). (**B**) Axial PET and CT scans (June 2019, March 2021, and August 2022).

**Figure 2:**
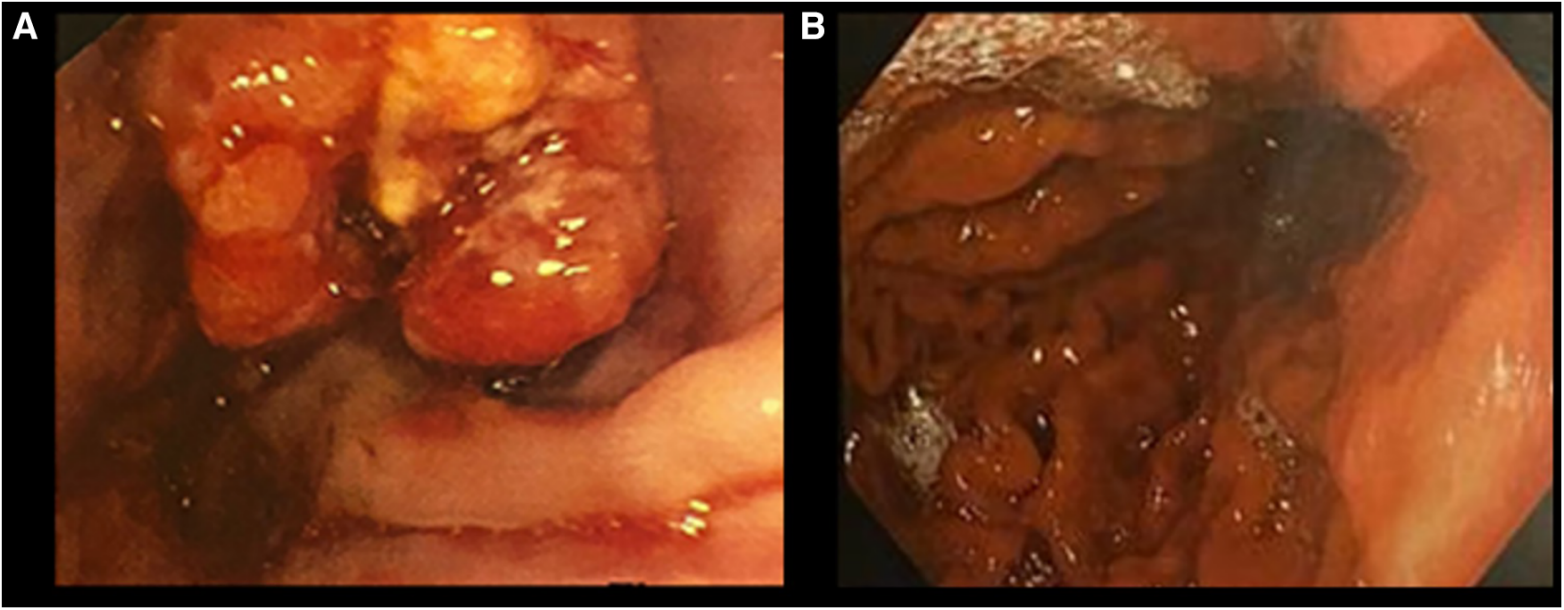
Endoscopic findings: (**A**) June 2019 and (**B**) May 2022.

Five months into chemotherapy, a follow-up PET scan revealed complete metabolic resolution of all previously metastatic areas, and another EGDS demonstrated a macroscopically clear esophagogastric junction (Figures 1, 2). Liver metastases were further re-evaluated using magnetic resonance imaging (MRI), which revealed a necrotic-colliquative aspect of the repetitive hepatic lesions, with the largest one in segment VIII.

Genetic mutational status was also assessed, and DNA extracted from selected neoplastic tissue samples underwent sequencing. The sequencing was performed by a targeted next-generation sequencing (NGS) panel (Agilent Technologies, Inc., Santa Clara, CA, USA) on the Illumina MySeq platform. DNA was quantitatively and qualitatively evaluated using the QIAxcel DNA High Resolution Kit on QIAxcel Advanced (Qiagen): quality and quantity were judged suitable for the requested analysis. A pathogenic mutation in the TP53 gene (R123W; Arg123Trp) was the only alteration found.

**Figure 3:**
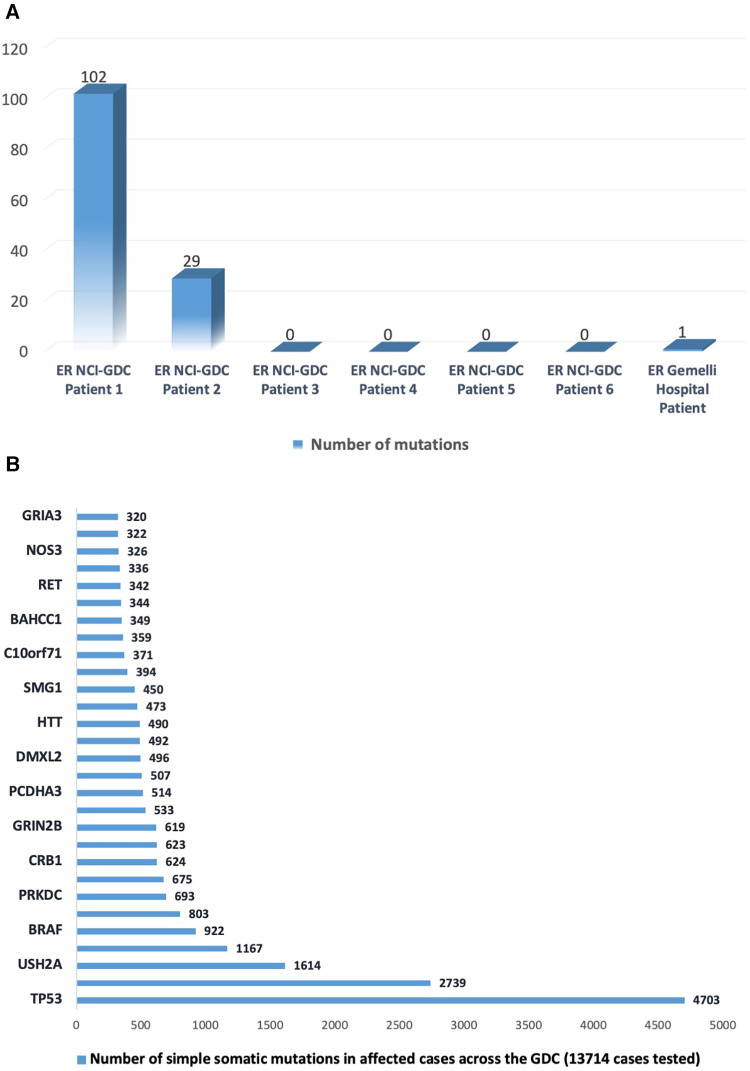
(**A**) Number of somatic mutations in the National Cancer Institute's Genomic Data Commons data portal (https://portal.gdc.cancer.gov/projects/EXCEPTIONAL_RESPONDERS-ER) in six patients and in the case reported at Gemelli Hospital. (**B**) Number of somatic mutations in the top 30-mutated genes as reported in the data portal (https://portal.gdc.cancer.gov).

This finding was compared with the data from the National Cancer Institute's (NCI's) Genomic Data Commons (GDC), which collects gene mutations from around 13,714 cases (10). The comparison revealed that TP53 was the most frequent simple somatic mutation involved in gastric cancer exceptional responders (Figure 3).

**Figure 4:**
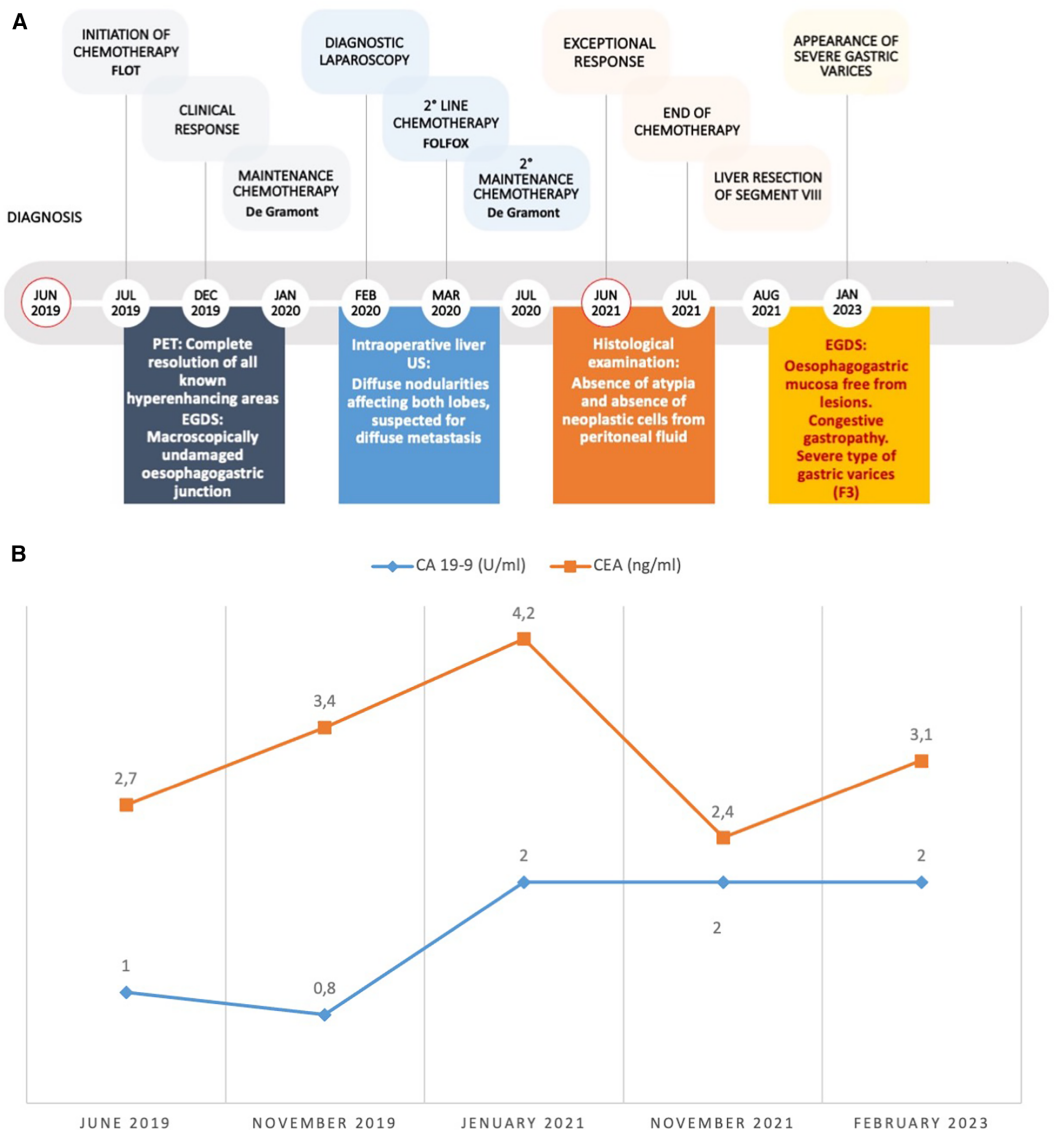
(**A**) Disease timeline and clinical course. Red circles mark the beginnings of medical history and the achievement of the exceptional clinical response. (**B**) time course of oncological markers: CA 19-9 (normal range < 35 U/ml) and CEA (normal range < 3 ng/ml). CEA, Carcino-Embryonic Antigen.

In February 2020, the patient underwent laparoscopic staging, which showed an absence of peritoneal carcinomatosis and no tumoral evidence at the esophagogastric junction. Intraoperative liver ultrasonography detected diffuse nodularity affecting both lobes. The cytological examination of peritoneal washing was negative for neoplastic cells. Consequently, the patient continued with five cycles of chemotherapy using folinic acid, fluorouracil, and oxaliplatin (FOLFOX), which had to be suspended four months later owing to its toxicity (Figure 4). At this stage, a CT scan revealed a further modest reduction in size of all liver lesions and the patient resumed the De Gramont scheme.

Following a new liver MRI, which indicated possible persistence of disease, a laparoscopic liver resection of segment VIII was performed. However, the histological examination of the resected specimen revealed the absence of neoplastic cells, indicating complete regression.

A subsequent EGDS, conducted in February 2023, confirmed an esophagogastric junction free from lesions and revealed severe gastric varices (F3), requiring two subsequent endoscopic ligation treatments (in March and July 2023). An abdominal ultrasound in July 2023 showed splenomegaly (spleen diameter: 17.6 cm) and signs of portal hypertension.

Furthermore, recent blood tests revealed a low white blood cell count and platelet count (2.82 and 69 × 10^9 ^/L, respectively), creatinine 1.37 mg/dl (normal range < 1.17 mg/dl), and the following hepatic enzymes and cholestasis indexes: transaminases AST 36 UI/L (normal range < 34 UI/L), transaminases ALT 30 UI/L (normal range < 49 UI/L), total bilirubin 3.6 mg/dl (normal range < 1.2 mg/dl), direct bilirubin 0.8 mg/dl (normal range < 0.3 mg/dl), gamma-glutamyl transferase 105 UI/L (normal range < 73 UI/L), and alkaline phosphatase 96 UI/L (normal range < 116).

Curiously, the blood values of tumor markers have remained within the normal range over time (Figure 4).

At 4 years and 3 months from diagnosis, and after a cumulative total of 33 cycles of chemotherapy, all CT and PET scans confirmed the exceptional response, with no metabolic activity observed in all body regions (Figures 1, 4).

To measure the patient's physical, psychological, and social functions, he completed the European Organization for Research and Treatment of Cancer (EORTC) Core Quality of Life questionnaire (EORTC QLQ-C30), showing optimal results, as reported in Table 1 (11).

**Table 1:** EORTC QLQ-C30 questionnaire (version 3.0): patient's reported outcomes and reference values in target population.

	All cancer patients: males^a^	All cancer patients: <50 years^a^	Esophageal cancer: <50 years^a^	Patient
Global health status/QoL			N/A	83.3
Mean (SD)	62.9 (23.8)	61.4 (23.4)		
Median (IQR)	66.7 (50–83.3)	66.7 (50–83.3)		
Physical functioning			N/A	93.33
Mean (SD)	78.5 (23)	80.2 (20.8)		
Median (IQR)	86.7 (66.7–100)	86.7 (66.7–100)		
Role functioning			N/A	100.0
Mean (SD)	73.4 (32.4)	68.6 (31.7)		
Median (IQR)	83.3 (50–100)	66.7 (50–100)		
Emotional functioning				83.3
Mean (SD)	73.9 (23.6)	69.2 (24.4)	62.0 (26.6)	
Median (IQR)	75 (58.3–91.7)	75 (58.3–91.7)	66.7 (50–83.3)	
Cognitive functioning				83.3
Mean (SD)	83.7 (21.1)	82.9 (21.6)	85.6 (21.8)	
Median (IQR)	83.3 (66.7–100)	83.3 (66.7–100)	100 (83.3–100)	
Social functioning				100.0
Mean (SD)	76.3 (28.4)	72.1 (29.5)	73.9 (29.9)	
Median (IQR)	83.3 (66.7–100)	83.3 (50–100)	83.3 (50–100)	
Fatigue				33.3
Mean (SD)	32.4 (27.4)	33.9 (26.1)	34.1 (22.5)	
Median (IQR)	33.3 (11.1–44.4)	33.3 (11.1–55.6)	33.3 (22.2–44.4)	
Nausea and vomiting				0.0
Mean (SD)	7.7 (17.2)	9.4 (19.1)	15.0 (21.7)	
Median (IQR)	0 (0–0)	0 (0–16.7)	0 (0–33.3)	
Pain				16.6
Mean (SD)	25.4 (29.6)	27.2 (28.8)	33.9 (25.8)	
Median (IQR)	16.7 (0–33.3)	16.7 (0–50)	33.3 (16.7–50)	
Dyspnea				0.0
Mean (SD)	21.1 (28.4)	17.1 (25.8)	13.9 (21.4)	
Median (IQR)	0 (0–33.3)	0 (0–33.3)	0 (0–33.3)	
Insomnia				0.0
Mean (SD)	26.7 (31.3)	30.2 (32.2)	36.7 (36.4)	
Median (IQR)	33.3 (0–33.3)	33.3 (0–66.7)	33.3 (0–66.7)	
Appetite loss				0.0
Mean (SD)	19.2 (30.2)	19.7 (29.1)	30.9 (35.8)	
Median (IQR)	0 (0–33.3)	0 (0–33.3)	33.3 (0–66.7)	
Constipation				0.0
Mean (SD)	16.2 (27.7)	15.3 (26.5)	22.0 (28.4)	
Median (IQR)	0 (0–33.3)	0 (0–33.3)	0 (0–33.3)	
Diarrhea				33.3
Mean (SD)	8.7 (20)	9.0 (19.9)	6.4 (17.2)	
Median (IQR)	0 (0–0)	0 (0–0)	0 (0–0)	
Financial difficulties				0.0
Mean (SD)	15.6 (27.9)	23.6 (32)	21.2 (30.2)	
Median (IQR)	0 (0–33.3)	0 (0–33.3)	0 (0–33.3)	

QoL, Quality of Life; IQR, interquartile range.

^a^ As reported in https://www.eortc.org/app/uploads/sites/2/2018/02/reference_values_manual2008.pdf.

## Discussion

In Western countries, CA is frequently diagnosed in advanced stages due to its non-specific symptoms and the lack of systematic screening policies.

Surgical resection with extended lymph node dissection represents the sole curative therapeutic option for resectable cases (5). However, at the time of diagnosis, only 40% of patients are eligible for surgery (11).

Despite advances in perioperative treatments and targeted therapy, the prognosis for CA remains poor.

The current 5-year survival rate for CA is only 10%–20%, with a median overall survival (OS) of just 1 year in cases of metastatic disease (12).

The response to chemotherapy varies depending on multiple factors and, in rare instances, becomes exceptional for reasons that are still not entirely clear (13).

Although HER2 overexpression is relatively uncommon in CA (ranging from 12% to 22%), it represents one of the primary therapeutic targets owing to the availability of anti-HER2 monoclonal antibody-based agents (14).

The FLOT or FOLFOX scheme is currently the recommended first-line therapy to improve the OS in cases of locally advanced HER2-negative CA (6, 15). In addition, high microsatellite instability (MSI-H) and Mismatch Repair deficient (dMMR) tumors appear to be favorable prognostic factors (16), both in terms of nodal status and downstaging (17, 18).

This patient represents an extremely rare case of an exceptional response to treatment for metastatic HER2-negative CA, in the absence of MSI-H and dMMR, treated with FLOT and de Gramont schemes.

Thanks to its excellent response to medical therapy, there has been no need so far for preoperative radiotherapy or surgical treatment on the primary tumor.

Notably, the only mutation detected through NGS was curiously TP53 (R123W; Arg123Trp), which is consistent with the profile of gastric cancer exceptional responders described in NCI's GDC.

However, it is important to note that this remarkable response to treatment resulted in severe post-chemotherapy liver disease, as evidenced by the discovery of liver nodularity during diagnostic laparoscopy, the development of F3 varices that necessitated endoscopic ligation, and suboptimal blood test results.

It is also crucial to note a significant “misleading” aspect in the liver MRI, after chemotherapy, suggesting a possible persistence of the disease. The discrepancy between MRI detection and the histopathological examination raises important questions about the reliability and accuracy of the diagnostic methods used, underlining in these cases the need for further investigation and consideration of alternative diagnostic approaches.

According to the NCI's GDC (10), an excellent response to chemotherapy for patients with stomach and gastroesophageal junction carcinoma may be achieved regardless of the number and type of gene mutations. Therefore, it is necessary to collect a substantial amount of data to identify patients eligible for an excellent response to systemic therapy.

## Data Availability

The datasets presented in this article are not readily available because of ethical and privacy restrictions. Requests to access the datasets should be directed to the corresponding author.
